# Data from proteomic analysis of the skin of Chinese giant salamander (*Andrias davidianus*)

**DOI:** 10.1016/j.dib.2015.02.010

**Published:** 2015-02-26

**Authors:** Xiaofang Geng, Hong Wei, Haitao Shang, Minghui Zhou, Bing Chen, Fuchun Zhang, Xiayan Zang, Pengfei Li, Jingyan Sun, Jing Che, Yaping Zhang, Cunshuan Xu

**Affiliations:** a State Key Laboratory Cultivation Base for Cell Differentiation Regulation and Henan Bioengineering Key Laboratory, Henan Normal University, Xinxiang 453007, China; b Department of Laboratory Animal Science, Third Military Medical University, Chongqing 400038, China; c Chongqing Kui Xu Biotechnology Incorporated Company, Kaixian Country, Chongqing 405423, China; d Xinjiang Key Laboratory of Biological Resources and Genetic Engineering, College of Life Science and Technology, Xinjiang University, Urumqi 830046, China; e State Key Laboratory of Genetic Resources and Evolution, and Yunnan Laboratory of Molecular Biology of Domestic Animals, Kunming Institute of Zoology, Chinese Academy of Sciences, Kunming 650223, China

## Abstract

The Chinese giant salamander (*Andrias davidianus*), renowned as a living fossil, is the largest and longest-lived amphibian species in the world. Its skin is rich in collagens, and has developed mucous gland which could secrete a large amount of mucus under the scraping and electric stimulation. The molting is the degraded skin stratum corneum. To establish the functional skin proteome of Chinese giant salamander, two-dimensional gel electrophoresis (2DE) and mass spectrometry (MS) were applied to detect the composition and relative abundance of the proteins in the skin, mucus and molting. The determination of the general proteome in the skin can potentially serve as a foundation for future studies characterizing the skin proteomes from diseased salamander to provide molecular and mechanistic insights into various disease states and potential therapeutic interventions. Data presented here are also related to the research article “Proteomic analysis of the skin of Chinese giant salamander (*Andrias davidianus*)” in the Journal of Proteomics [1].

**Specifications table**

**Table:** 

Subject area	Biology
More specific subject area	Giant salamander skin proteomics
Type of data	Table, excel file
How data was acquired	2-D electrophoresis (GE Healthcare, USA)
	ImageScanner III Labscan (GE Healthcare, USA)
	MALDI-TOF/MS and Biotools (Bruker Daltonics, Germany)
	MASCOT search engine (Matrix Science, London, UK)
	uniprot database
Data format	Analyzed
Experimental factors	Skin, mucus and molting samples from Chinese giant salamander were collected and used to systematically characterize the proteome
Experimental features	2-D electrophoresis coupled with MALDI-TOF/MS
Data source location	Xinxiang, Henan Province, China
Data accessibility	Analyzed datasets are directly provided with this article

**Value of the data**

• The data provide new insight in the aspects of the proteomes in the skin, mucus and the molting of Chinese giant salamander.
• The data gathered after bioinformatics analysis provide the potential physiological functions of the identified proteins.
• The data can potentially serve as a foundation for future studies characterizing the skin proteomes from diseased salamander or from other amphibian species.

## 1 Data, experimental design, materials and methods

### 1.1 Animals

Three or four years old male healthy Chinese giant salamanders with body length 60–100 cm and weight about 3 kg, were obtained from a giant salamander breeding base in Wen Quan Zhen, Kaixian Country, Chongqing Municipality, China. The molting was obtained from the breeding pool of Chinese giant salamanders. The mucus was secreted by the dorsal skin of Chinese giant salamanders under the scraping stimulation with a triangle, and was collected in a sterile tube. Subsequently, they were anesthetized and sacrificed by decapitation. The skin tissues were immediately dissected from the giant salamanders, and washed in sterile PBS. The skin, mucus and molting samples were stored in liquid nitrogen for further use. All experiments were performed in strict accordance with the Animal Protection Law of China.

### 1.2 Protein extraction and two-dimensional gel electrophoresis (2D)

Protein extraction was performed according to the previously described method [2]. Briefly, the skin, mucus and molting samples were respectively grinded into fine powder in liquid nitrogen, and then were suspended in lysis buffer (30 mM Tris, 7 M urea, 2 M thiourea, 4% CHAPS). The suspension was mixed at 4 °C for 1 h by vortex, and then was centrifuged at 20,000 g for 1 h. The supernatant was collected and stored at −80 °C for further use. The concentration of total proteins was determined using a 2D Quantification kit (GE Healthcare, USA).

2D was performed according to operating manual (GE Healthcare, USA), and each sample was repeated three times. In brief, 1000 µg of samples were dissolved in 450 µL rehydration buffer (7 M urea, 2 M thiourea, 2% CHAPS, 18 mM DTT, 2% IPG buffer 3–10 NL). Then IPG gel strip (pH 3–10 NL, 24 cm) were rehydrated for 12 h at 20 °C. The isoelectric focusing (IEF) was performed in Ettan IPGphor (GE Healthcare, USA) following the conditions: ramped to 250 V in 1 h, held at 1000 V for 3 h, ramped to 10,000 V in 3 h, and held at 10,000 V for 8 h. After IEF, each gel strip was equilibrated for 15 min in 15 mL equilibration buffer (50 mM TrisHCl pH 8.8, 6 M urea, 30% glycerol, 2% SDS, 1% DTT) and subsequently alkylated for 15 min in alkylation equilibration buffer (50 mM TrisHCl pH 8.8, 6 M Urea, 30% v/v glycerol, 2% SDS, 2.5% iodoacetamide). The second dimension SDS-PAGE was performed in an Ettan DALT Six electrophoresis device (GE Healthcare, USA) according to the following procedures: 1 h at 1w per gel followed by 13w per gel until the bromophenol blue reached the bottom of the gel. Finally, the generated gels were stained with Coomassie brilliant blue G250. All the gels were scanned to obtain the images using a scanner (GE Healthcare, USA) according to the manufacturer׳s protocol. The abundance of each protein spot in scanned images was quantitatively analyzed using Image Master Platinum 7.0 software (GE Healthcare, USA). The protein spot detected in at least two replicates of each sample was used to be further identified by mass spectrometry.

### 1.3 Protein identification by mass spectrometry

The protein spots in the stained gels were manually excised, decolorized, dehydrated and whitened according to the instruction and operation manual (Bruker, Germany). In-gel digestion was performed firstly with 2 µl of 0.01 μg/μl trypsin solution at 4 °C for 30 min, and then with 5 µl of 25 mM NH4HCO3 at 37 °C overnight. 1 µL of digestion sample was placed on MTP AnchorChip^™^ 800/384 sample target, and then 1 µL 0.4 mg/ml HCCA solution was added. External mass calibration was performed by using peptide standards with mass range 700–3200 Da (Bruker Dalton, Germany). The protein was identified by Matrix assisted laser desorption ionization time-of-flight mass spectrometry (MALDI-TOF/MS) (Autoflex III Brucker Daltonics, Germany). The parameters of MALDI-TOF/MS were set as follows: reflection mode, protein mass *m*/*z* 700–4200, trypsin as the digestion enzyme with one cleavage site, fixed modifications carbamidomethyl (C), variable modifications oxidation (M), peptide mass tolerance ±100 ppm, fragment mass tolerance ±0.5 Da, signal–noise ratio (SNR) of MS and MS/MS with 30 and 20 respectively, number of matched peptide fragments at least 3 pieces. The retrieval of peptide fragment was preformed if mass spectral peaks *m*/*z*≥600. The selected peptides were analyzed using BioTools (version 3.2, Bruker Daltonics, Germany), and then were searched against Swiss-Prot database with taxonomy Andrias, Cryptobrachidae, Caudata, Amphibia database using MASCOT search engine (Matrix Science Ltd., version 2.1), if not found, database of danio rerio, Rattus, Mus musculus and Homo sapiens were searched in order. Only those proteins identified by MASCOT search criteria with the top significant scores were considered as acceptable (see Supplementary Table 1).

### 1.4 Bioinformatics analysis

The protein names were firstly translated into gene names, and then were uploaded to Gene Ontology (GO) annotation website (http://geneontology.org/) to obtain biological functions (see Supplementary Table 2). In order to evaluate the similarities or differences of protein expression profiles among different tissues, Cluster 3.0/TreeView software was used for hierarchical clustering of the identified proteins [3]. The Cluster organizes and analyzes the data in a number of different ways. TreeView allows the organized data to be visualized and browsed. In this study, the intensities of the identified proteins were directly used for hierarchical clustering, and the missing values were treated as zero. The parameters array cluster and correlation (uncentered) were used in this analysis. Then complete linkage clustering was selected to analyze these data. Moreover, a Venn diagram was constructed to identify common and exclusively proteins in the three samples (see Fig. 2 in Ref [1]).

## Footnotes

Appendix A Supplementary data associated with this article can be found in the online version at doi:10.1016/j.dib.2015.02.010.
